# Supplementary material for: Deep brain stimulation of the internal capsule enhances human cognitive control and prefrontal cortex function
Source: Nat Commun. 2019 Apr 4;10:1536. doi: 10.1038/s41467-019-09557-4 (PMC6449385; doi:10.1038/s41467-019-09557-4)
Supplement: Supplementary file 1 — Supplementary Information [file 41467_2019_9557_MOESM1_ESM.pdf]

Deep Brain Stimulation of the Internal Capsule Enhances Human Cognitive Control and Prefrontal Cortex Function

Widge et al.

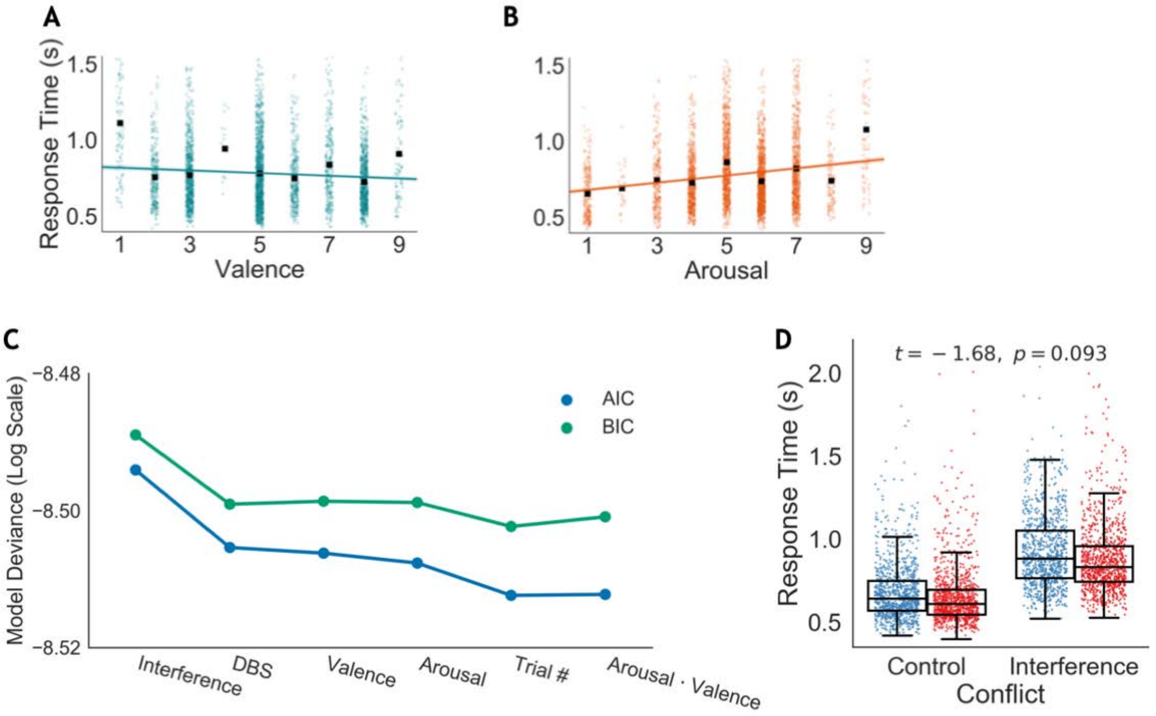

Supplementary Figure 1: Effects of emotional distractors and conflict during affective version of the Multiple Source Interference Task.

(**A**), negative emotional valence caused a mild slowing and positive pictures a mild quickening, for a mean difference of 72 ms on the most positive/negative pictures compared to neutral (t=-2.01, p<0.044 for Wald test of regression coefficient in the generalized linear model). Colored points represent individual trials' RT, black points represent mean RT at each rating level, and lines show best linear fit from a robust regression.

(**B**), emotional arousal had stronger effects on response times than valence, with a predicted slowing of 59 ms per step of the 9-point IAPS scale (t=4.5, p<7.1e-6, Wald test of regression coefficient). Valence and arousal are subjects' individualized, self-reported emotional ratings of the images they actually observed during the task.

(**C**), behavioral model validation. Curves show Akaike's Information Criterion (AIC) and the Bayesian Information Criterion (BIC) for sequential addition of each independent predictor variable to the gamma-distribution generalized linear model (GLM) used to model response time. Interaction terms either increased the information criterion (Arousal x Valence as shown, DBS x Arousal, DBS x Valence), or marginally lowered the criterion but yielded estimated coefficients whose confidence interval included zero (DBS x Interference, Arousal x Interference). Thus, the behavioral analyses used all main effect terms shown here but no interaction terms. The EEG analysis included the DBS x Interference term on the basis of a pre-specified hypothesis, although no effects were ultimately found for that interaction term.

(**D**), example plot of an interaction term without a significant regression coefficient, the DBS x Interference interaction. The ON-OFF difference is present and is about the same size in both the Interference and Control trial types (interaction coefficient estimate -0.031 to 0.002, p=0.093).

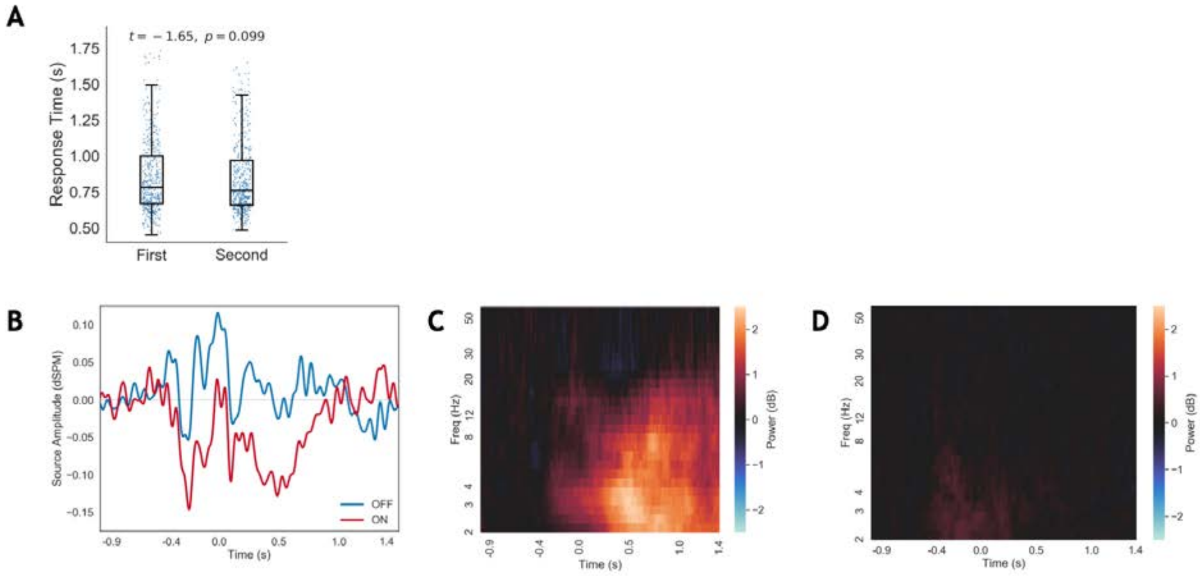

Supplementary Figure 2: Manipulation checks.

(**A**), repeated performance of MSIT, in the absence of a DBS intervention but in the presence of intracranial electrodes, does not produce response time changes. 8 subjects undergoing intracranial monitoring for epilepsy performed two or more blocks of MSIT spaced by 18-260 minutes (mean spacing, 88 minutes, comparable to the primary DBS study). In a GLM analysis equivalent to that used in main text Figure 1, there was no significant effect of the first vs. second block (t=1.65, p=0.099). Error bars denote standard error of the mean (SEM).

(**B**), demonstration of oscillatory activity after source localization. Shown is the grand average source time course of the anterior IFG label (the same label plotted in main text Figure 1C), time-locked to MSIT stimulus onset at 0 s. Positive- and negative-going components, with sinusoidal oscillations superimposed on the ERP, are visible.

(**C**), Morlet wavelet time-frequency representation of the same source label and time window as in (B). This panel shows the non-phase-locked activity, i.e. the oscillations analyzed in this paper, normalized against the pre-trial baseline. An increase in low-frequency oscillatory power is visible after the stimulus onset.

(**D**), Morlet wavelet time-frequency representation as in (C), but of the phase-locked activity. Very little change is visible, in theta or any other frequency band, compared to the non-phase-locked activity.

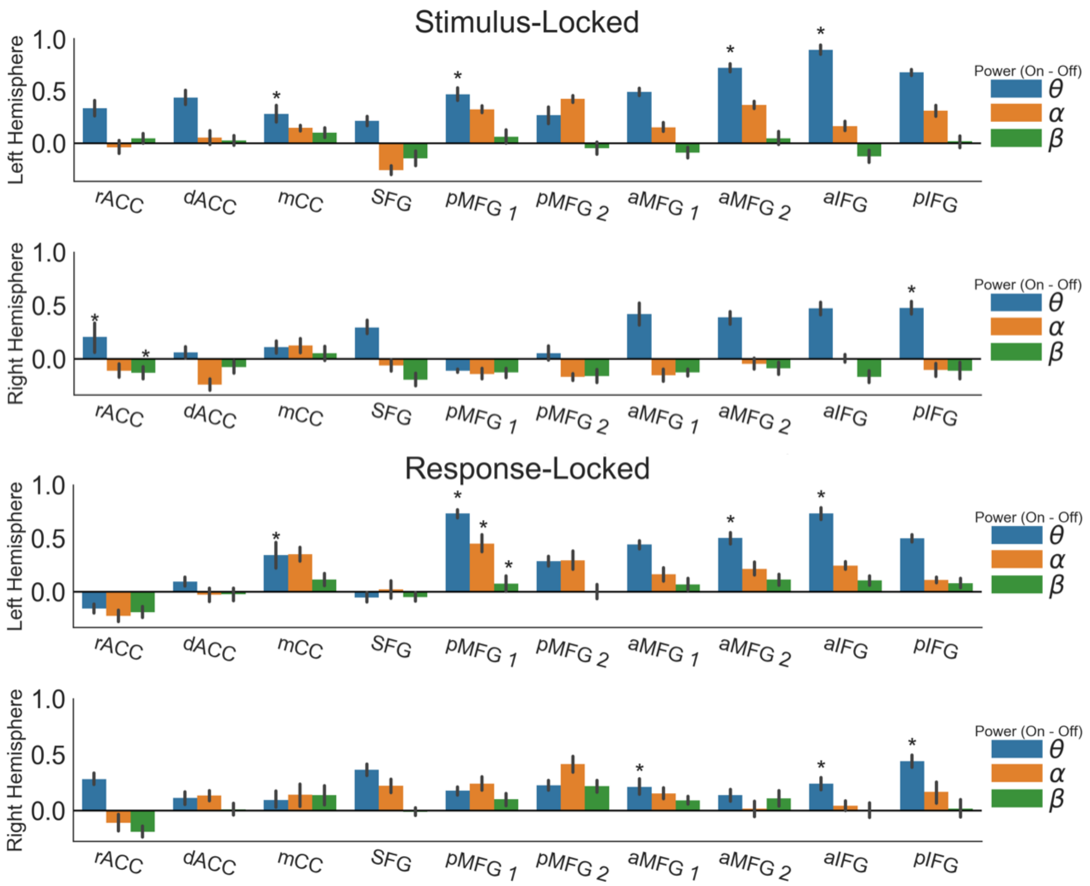

Supplementary Figure 3: DBS ON-OFF power change in theta (4-8 Hz), alpha (8-15 Hz), and beta (15-30 Hz) across all tested labels, in stimulus- (MSIT onset) and response-locked analyses. Each bar shows the mean ON-OFF change (expressed as dB from baseline) from 0 to 400 ms after the MSIT onset (stimulus-locked) or -200 to +200 ms around the response (response-locked). These are the same time windows used in main text Figures 3A-B and 3E-F. Error bars denote bootstrapped confidence intervals of the theta power change, calculated from 1,000 resamplings with replacement. Asterisks denote label/frequency combinations where there was a $p<0.05$ significant temporal cluster for at least part of the trial, which may not necessarily overlap with the windows plotted here. As with the main text analysis, the cluster p-values were false-discovery-rate corrected for testing of multiple labels and frequency bands.

There were significant ON-OFF theta changes in multiple labels, as also reflected in main text Figure 3G. These favored the left hemisphere, but were visible in some right-sided labels, particularly inferior frontal gyrus (IFG). Other frequency bands, however, show no significant DBS-induced change. The sole exception is the posterior middle frontal gyrus (pMFG 1) in the response-locked analysis. In that label, both alpha and beta band power increased with DBS ON. Those changes, however, are much smaller than the corresponding theta-band changes, suggesting that overall the primary effect of VCVS DBS is on task-induced theta oscillations.

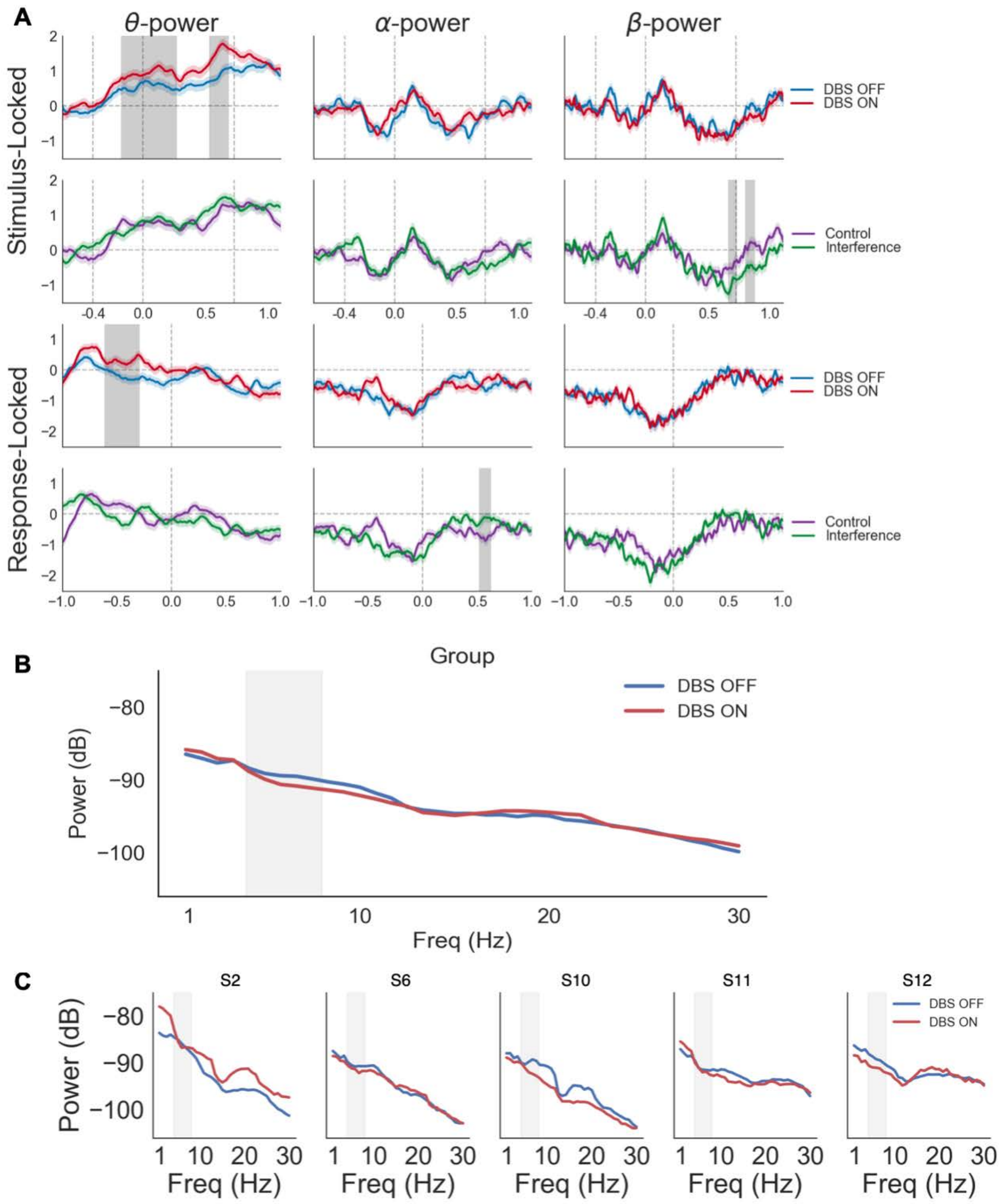

Supplementary Figure 4: Sensor-space results in the theta band.

(**A**), DBS and interference effects at the Fz sensor, which was at the center of the task-driven theta-band increase shown in main text Figure 3A. Performing MSIT increased theta power over

baseline, and that increase was augmented by DBS ($p<0.05$ cluster significance after FDR correction for multiple frequency bands): clusters at -224 to 268 ms and 529-683 ms stimulus-locked and -614 to 295 ms response-locked. No such changes are present in alpha or beta bands, similar to the source-space results of Supplementary Figure 3. Theta-band Interference effects were not significant at this sensor, although we do see small clusters of alpha- and beta-band change.

(**B**), grand-mean eyes-open resting-state spectrum ($n=5$) at sensor Fz, calculated from 60 non-overlapping 1-second epochs in each subject. Resting-state theta does not change significantly (Mann-Whitney $U = 5.00$, $p = 0.072$) between ON and OFF conditions. Shaded area marks the boundaries of the theta band.

(**C**), individual subjects' eyes-open resting state spectra at sensor Fz, calculated as in (B). The theta band is highlighted with a shaded background. Resting theta power is increased with DBS OFF in 2/5 subjects, essentially unchanged in 3/5, and decreased in none. Subject labels correspond to rows in Table 1.

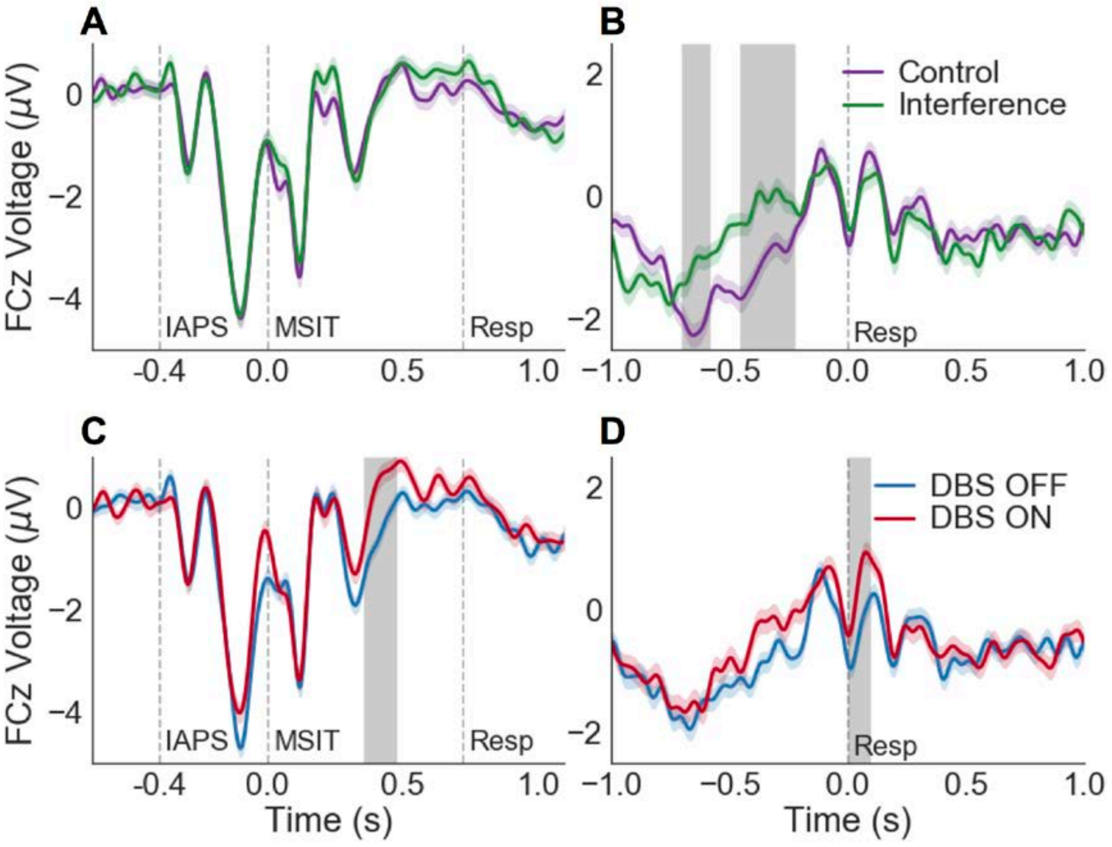

Supplementary Figure 5: Interference effects on ERPs at sensor FCz, which lies in the center of the negativity seen in main text Figure 4A-B.

Interference (A-B) and DBS (C-D) event-related potential (ERP) effects as recorded directly at midline frontal scalp sensor (FCz). Plotting conventions are as in Figures 3-4, and grey shading again reflects $p<0.05$ significant clusters. No FDR correction is applied, as we tested only this single sensor in the sliding regression analysis.

Similar to other reports [1,2], MSIT induces sharp negative-going peaks in this fronto-central sensor in a stimulus-locked analysis (A,C). Response-locked analyses similarly resemble other conflict tasks, e.g.[1].

Interference effects reached pre-specified statistical significance thresholds in the response-locked analysis before the response (B, $p<0.001$), but not in the stimulus-locked analysis at this sensor (A). DBS slightly altered ERPs in both stimulus- (C, $p=0.024$) and response-locked (D, $p=0.024$) regressions. These effects could not be attributed to any single label in the source-localized ERP analysis (main text Figure 4), possibly because they arise from the summation of phase-locked activity in multiple cortical generators. These DBS effects on the sensor-space ERP were much smaller than the effects on source-space theta oscillations, in both their absolute magnitude and their temporal extent (see main text Figure 3G).

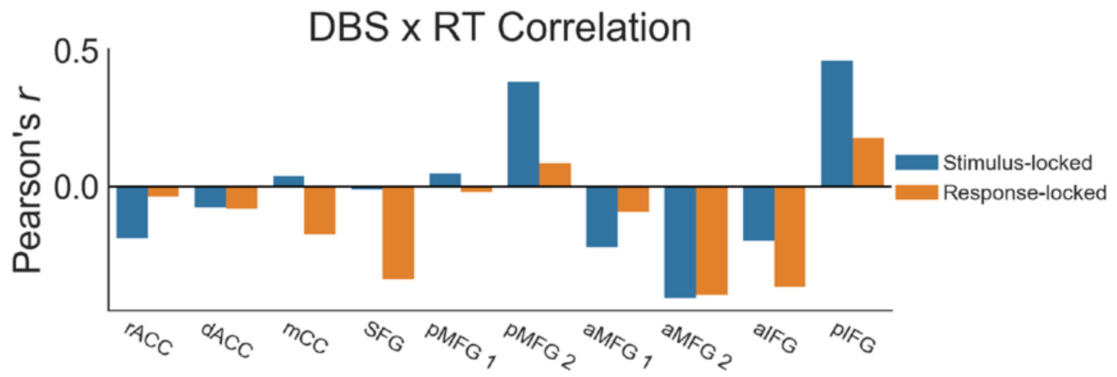

Supplementary Figure 6: DBS-induced changes in response time (RT) correlate with DBS-induced changes in task-related theta power. Each pair of bars shows, in one source-localization label, the correlation between each subject's RT change (ON-OFF) from DBS and theta power change in that label. Negative correlations thus denote labels where increased theta power (a putative marker of increased top-down control) correlates with faster RTs. Because the significant clusters had different temporal spans in each label, and because some labels had no significant theta change, we computed these correlations using a standard time window across clusters. The stimulus-locked correlation extracted theta power from 0 to 400 ms after the MSIT onset; the response-locked correlation extracted theta from -200 to +200 ms around the response. These are the same time windows used in Figure 3 and in Supplementary Figure 3. Despite using windows not designed to optimally extract the theta change in any given label, we observed potentially meaningful correlations (absolute magnitude above 0.25) in half our tested labels.

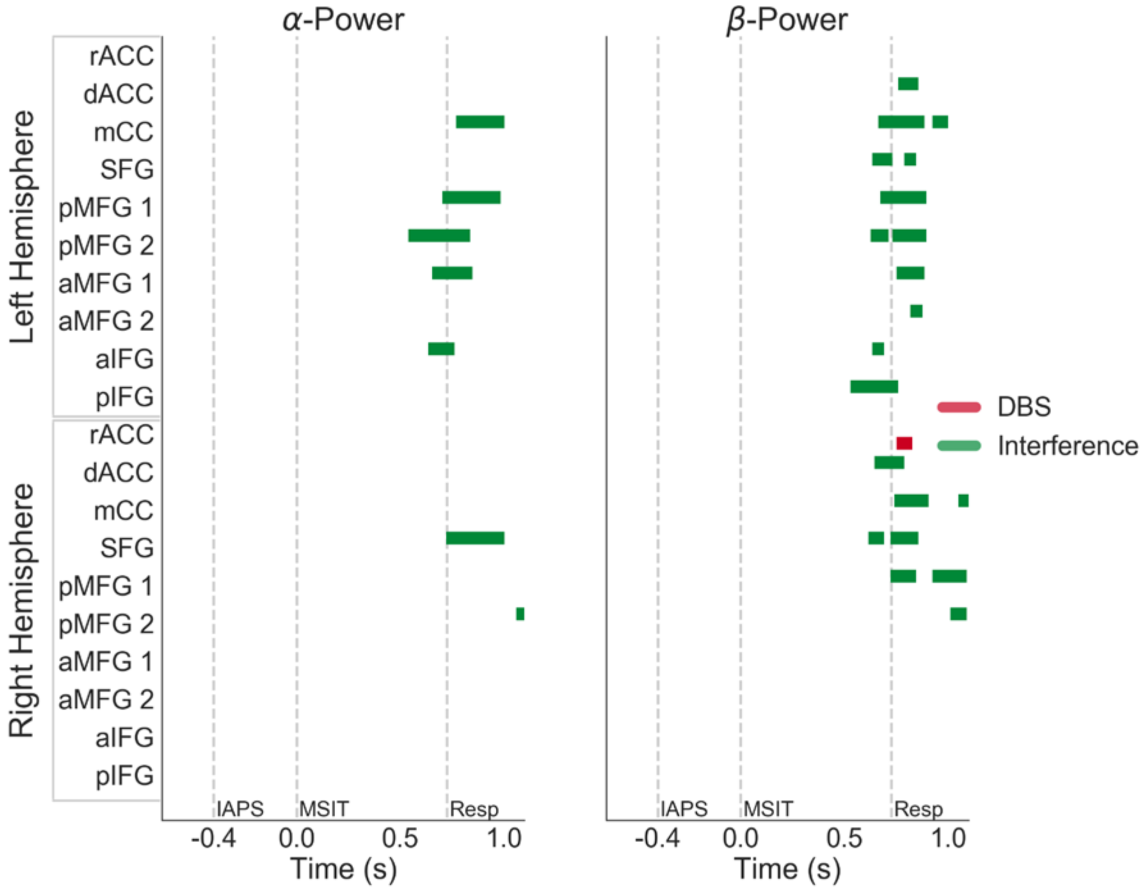

Supplementary Figure 7: Task and DBS related variable encoding in alpha and beta bands. Colors and plotting conventions follow main text Figure 3G: each colored bar represents the temporal extent of a $p<0.05$ (after FDR correction) significant cluster. DBS does not significantly modulate either band, with the exception of a small post-response cluster in right rostral anterior cingulate (rACC). Given its relation to the response time and given that this cluster is in the beta band, which often reflects movement preparation and execution, we attribute this to differential motor timing in the ON vs. OFF condition.

Similarly, all significant alpha- and beta-band effects of Interference occur around the mean response time. This matches the sensor-space results of González-Villar et al.[2] As with DBS, the Interference/Control trials have very different response times between the conditions, and we attribute these peri-response effects to that time shift.

**Label Name**	**Lausanne Label**
dACC	caudalanteriorcingulate_1-lh
dACC	caudalanteriorcingulate_2-lh
dACC	caudalanteriorcingulate_1-rh
dACC	caudalanteriorcingulate_2-rh
dACC	caudalanteriorcingulate_3-rh
pMFG 1	caudalmiddlefrontal_1-lh
pMFG 1	caudalmiddlefrontal_5-lh
pMFG 1	caudalmiddlefrontal_6-lh
pMFG 1	caudalmiddlefrontal_1-rh
pMFG 1	caudalmiddlefrontal_2-rh
pMFG 1	caudalmiddlefrontal_5-rh
pMFG 2	caudalmiddlefrontal_2-lh
pMFG 2	caudalmiddlefrontal_3-lh
pMFG 2	caudalmiddlefrontal_4-lh
pMFG 2	caudalmiddlefrontal_4-rh
pMFG 2	caudalmiddlefrontal_3-rh
aMFG 1	rostralmiddlefrontal_2-lh
aMFG 1	rostralmiddlefrontal_3-lh
aMFG 1	rostralmiddlefrontal_2-rh
aMFG 1	rostralmiddlefrontal_3-rh

aMFG 2	rostralmiddlefrontal_5-lh
aMFG 2	rostralmiddlefrontal_1-lh
aMFG 2	rostralmiddlefrontal_1-rh
aMFG 2	rostralmiddlefrontal_5-rh
aIFG	parsopercularis_2-lh
aIFG	parstriangularis_2-lh
aIFG	parstriangularis_1-rh
aIFG	parsopercularis_1-rh
pIFG	parsopercularis_3-lh
pIFG	parsopercularis_4-lh
pIFG	parsopercularis_3-rh
pIFG	parsopercularis_4-rh
SFG	caudalmiddlefrontal_1-lh
SFG	caudalmiddlefrontal_1-rh
mCC	posteriorcingulate_2-lh
mCC	posteriorcingulate_3-lh
mCC	posteriorcingulate_2-rh
mCC	posteriorcingulate_3-rh
rACC	rostralanteriorcingulate_1-lh
rACC	rostralanteriorcingulate_2-lh
rACC	rostralanteriorcingulate_1-rh
rACC	rostralanteriorcingulate_2-rh

Supplementary Table 1: Mapping between the 243-region Lausanne parcellation and the larger anatomic labels used for source localization in this paper. Each of our labels is made by merging 4-6 labels from the Lausanne atlas. We chose the labels to merge such that each region of interest for source localization would have approximately the same number of vertices in the cortical

surface mesh. Equalizing the label size makes the electrophysiologic results more closely comparable between labels.

Supplementary References

1. Cohen, M. X. & Donner, T. H. Midfrontal conflict-related theta-band power reflects neural oscillations that predict behavior. *J. Neurophysiol.* **110**, 2752–2763 (2013).
2. González-Villar, A. J. & Carrillo-de-la-Peña, M. T. Brain electrical activity signatures during performance of the Multisource Interference Task. *Psychophysiology* **54**, 874–881 (2017).
